# The Scion/Rootstock Genotypes and Habitats Affect Arbuscular Mycorrhizal Fungal Community in Citrus

**DOI:** 10.3389/fmicb.2015.01372

**Published:** 2015-12-01

**Authors:** Fang Song, Zhiyong Pan, Fuxi Bai, Jianyong An, Jihong Liu, Wenwu Guo, Ton Bisseling, Xiuxin Deng, Shunyuan Xiao

**Affiliations:** ^1^Key Laboratory of Horticultural Plant Biology (Ministry of Education), Key Laboratory of Horticultural Crop Biology and Genetic Improvement (Central Region, Ministry of Agriculture), College of Horticulture and Forestry Sciences, Huazhong Agricultural UniversityWuhan, China; ^2^Laboratory of Molecular Biology, Department of Plant Sciences, Wageningen UniversityWageningen, Netherlands; ^3^Department of Plant Science and Landscape Architecture, Institute for Bioscience and Biotechnology Research, University of MarylandRockville, MD, USA

**Keywords:** arbuscular mycorrhizal fungi (AMF), citrus, community structure, habitat, scion/rootstock genotype

## Abstract

Citrus roots have rare root hairs and thus heavily depend on arbuscular mycorrhizal fungi (AMF) for mineral nutrient uptake. However, the AMF community structure of citrus is largely unknown. By using 454-pyrosequencing of 18S rRNA gene fragment, we investigated the genetic diversity of AMF colonizing citrus roots, and evaluated the impact of habitats and rootstock and scion genotypes on the AMF community structure. Over 7,40,000 effective sequences were obtained from 77 citrus root samples. These sequences were assigned to 75 AMF virtual taxa, of which 66 belong to *Glomus*, highlighting an absolute dominance of this AMF genus in symbiosis with citrus roots. The citrus AMF community structure is significantly affected by habitats and host genotypes. Interestingly, our data suggests that the genotype of the scion exerts a greater impact on the AMF community structure than that of the rootstock where the physical root-AMF association occurs. This study not only provides a comprehensive assessment for the community composition of the AMF in citrus roots under different conditions, but also sheds novel insights into how the AMF community might be indirectly influenced by the spatially separated yet metabolically connected partner—the scion—of the grafted citrus tree.

## Introduction

Arbuscular Mycorrhizal Fungi (AMF) form mutualistic relationship with most terrestrial plants (Smith and Smith, 2011), and facilitate the uptake of water and nutrients such as phosphorus and nitrogen from soil by roots (Harrison et al., 2002; Govindarajulu et al., 2005). It has been well-established that AMF can promote growth of host plants, and enhance their tolerance to biotic and abiotic stresses (Porcel and Ruiz-Lozano, 2004; Pozo and Azcón-Aguilar, 2007; Pineda et al., 2010; Cameron et al., 2013).

Citrus is one of the most important fruit crops worldwide. Because citrus trees have short or even rare root hairs, it is believed that uptake of mineral nutrients from soil in citrus roots is largely dependent on symbiotic AMF (Davies and Albrigo, 1999). However, to date, there are only a few preliminary studies with regard to the genetic diversity of AMF associated with citrus roots (Nemec et al., 1981; Vinayak and Bagyaraj, 1990; Wang and Wang, 2014). The complete AMF community structure as well as possible impact of environmental factors and host genotypes on the AMF community composition in citrus roots remain to be explored.

Earlier studies of AMF genetic diversity were based on spore morphology and/or conventional DNA-based molecular methods. However, these methods have apparent limitations in resolving the genetic difference of AMF (Merryweather and Fitter, 1998; Öpik et al., 2009). In recent years, high-throughput DNA sequencing technologies have been widely used in determining the fungal community associated with plants (Peršoh, 2015). Similar approaches have also been taken to determine AMF communities from various natural or agricultural environments such as forestland, farmland, grassland and farming-pastoral ecotone (Öpik et al., 2009; Dumbrell et al., 2011; Lin et al., 2012; Hiiesalu et al., 2014; Xiang et al., 2014). These studies have shown that different environmental factors, including land use, fertilization, geographic location, season variation and host plant diversity, may have profound effects on their associated AMF community structures (Davison et al., 2012; Lin et al., 2012; Öpik et al., 2013; Hiiesalu et al., 2014; Xiang et al., 2014). Of particular note is that host plant genotypes (i.e., different plant species or even different crop cultivars) also seemed to have an impact on the genetic diversity of AMF based on some preliminary studies using spore morphology or Sanger sequencing of 18S rDNA fragments (Eom et al., 2000; Mao et al., 2014). Apparently, these results need to be substantiated by more compelling evidence from more in-depth studies using high-throughput DNA sequencing technologies.

To evaluate the possible effects of habitats and host genotypes on AMF community in citrus, we first assessed the genetic diversity of AMF by 454 pyrosequencing of the 18S ribosomal RNA (rRNA) gene fragments of AMF amplified from DNA samples prepared from citrus roots, and then we performed Principal component analysis (PCA) using the OTU abundance of each sample. Specifically, as the citrus trees are vegetatively propagated by grafting for commercial production, both the effects of their scion (the aboveground of a grafted tree) and rootstock (where root-AMF symbiosis occurs) on AMF community were further investigated.

## Materials and methods

### Root sample collection and processing

Citrus root samples were collected from eight citrus-producing areas in China (Figure S1, Supporting information), including Wuhan (30°34′ N, 114°18′ E), Yiling (30°58′ N, 111°7′ E), Danjiangkou (32°33′ N, 111°10′ E), Shaoyang (27°14′ N, 111°26′ E), Xunwu (24°54′ N, 115°39′ E), Xinfeng (25°16′ N, 114°58′ E), Chengdu (30°40′ N, 104°03′ E) and Hanzhong (33°04′ N, 107°02′ E). A total of 77 root DNA samples from citrus trees of 23 different genotypes or scion/rootstock combinations (i.e., sample groups) grown under different conditions (Table S1 and Figure S1, Supporting information). For details, seven samples were collected in Wuhan, four of which were citrus seedlings with different genotypes, i.e., (i) *Poncirus*, (ii) *Citrange* (*Citrus sinensis* × *Poncirus trifoliate*), (iii) an allotetraploid originated from cell fusion of *India lime* and *Sunki orange*, designated *Lime* × *Orange*, and (iv) a hybrid originated from hybridization between *Poncirus* and *Red tangerine*, designated *Poncirus* × *Tangerine*. The remaining three samples were collected from citrus trees with distinct scions grafted onto the same type of rootstock, i.e., (i) *Washington navel orange* (*Citrus sinensis*) grafted onto *Poncirus* (*Poncirus trifoliate*) designated *Orange*/*Poncirus*, (ii) *Mandarin* (*Citrus reticulate*) grafted onto *Poncirus*, designated *Mandarin*/*Poncirus*, and (iii) *HB pummelo* (*Citrus grandis*) grafted onto *Poncirus*) designated *Pummelo*/*Poncirus*. In Yiling, we collected three samples from citrus tree with three scion/rootstock combinations, i.e., *Ponkan* (*Citrus reticulata Blanco*)/*Citrange, Ponkan*/*Poncirus*, and *Mandarin*/*Poncirus*. In Dangjiangkou and Shaoyang, we collected one sample from citrus tree with *Mandarin*/*Poncirus*. In Xunwu, we collected three samples. Two of these three samples were collected from citrus trees with two different scions grafted on the same rootstock, i.e., *Newhall sweet orange/Poncirus and Mandarin/Poncirus*. The remaining one sample was collected from Huanglongbing (also known as citrus greening) infected citrus tree with the same genotype of *Mandarin/Poncirus*. In Xinfeng, two samples were collected from two different two rootstocks, *Poncirus* and *Red tangerine*, that were approach-grafted to the single scion (*Newhall sweet orange*), i.e., *Newhall sweet orange*/*Poncirus* and *Newhall sweet orange*/*Red tangerine* (see Figure S2, Supporting information). In Chengdu, three samples were collected from citrus trees with the same scions grafted onto distinct rootstocks, i.e., *Mandarin*/*Yuzu* (*Citrus junos*), *Mandarin*/*Poncirus*, and *Mandarin*/*Red tangerine* (*Citrus tangerinaHort*). In Hanzhong, three samples were collected. Two of these three samples were from the same *Newhall sweet orange*/*Poncirus* combination in two neighboring orchards, the other one from *Newhall sweet orange*/*Zhique* (*Citrus ichangensi* × *Poncirus trifoliate*). Each individual raw sample contained roots with surrounding soils collected from three different locations around one citrus tree. Each group of samples (representing one habitat with a particular scion/rootstock combination if applicable) consisted of at least three different citrus trees (i.e., replicated plots) within 10 m of each other. The tree locations, number of replicated plots and the tree genotypes for all the samples processed in this study were listed in Table S1.

The raw samples were processed according to a published protocol (Lundberg et al., 2012). Loose soils were removed from roots by gentle shaking, and lateral roots were collected from the raw samples with sterile gloves (sprayed with 70% Ethanol). Lateral roots were placed in a clean and sterile 50-ml tube containing 25-ml phosphate buffer (per liter: 6.33 g of NaH_2_PO_4_·H_2_O, 16.5g of Na_2_HPO_4_·7H_2_O, 200 μl Silwet L-77). The tube was vortexed at a maximum speed for 1 min to release most of the tightly attached soils. Then the lateral roots were transferred to a new tube containing 25-ml phosphate buffer and subjected to vortexing until the buffer was clear after vortexing. The roots were then washed in an ultrasonic cleaner for 10 min to remove the tiny soils and loose microbes on the root surface. After cleaning, lateral roots were frozen with liquid nitrogen, and stored at −80°C.

### DNA extraction and 454 pyrosequencing

DNA was extracted from 20 mg of homogenized citrus lateral roots using a CTAB method previously described (Cheng et al., 2003). In order to define the AMF community in citrus roots, we used 454 GX FLX pyrosequencing of amplicons of the small subunit region of ribosomal RNA gene (SSU rRNA). Two primer pairs NS31/AM1 and AMV4.5NF/AMDGR were used to estimate AMF community structure and abundance through 454 pyrosequencing (Öpik et al., 2009; Lumini et al., 2010; Dumbrell et al., 2011; Lin et al., 2012). A pre-experiment showed that the primer pair AMV4.5NF/AMDGR yielded more effective reads (reads with correct TCMID sequence and forward primer sequence; ≥200 bp in length) and more Glomeromycota sequences than the other primer pair with our DNA samples prepared from citrus roots (data not shown). Therefore, this primer pair was used in this study to amplify Glomeromycota sequences from all of our DNA samples with two different adaptors (underlined). A 10-bp multiplex identifier (TCMID; indicated by “NNNNNNNNNN”) is inserted between adaptor A and primer AMV4.5NF (italicized), resulting in the fusion forward primer 5′-GCCTCCCTCGCGCCATCAG-NNNNNNNNNN-*AAGCTCGTAGTTGAATTT CG*-3′, the reverse fusion primer is 5′-GCCTTGCCAGCCCGCTCAG-*CCCAACTATCCCTATTAA TCAT*-3′, with the primer AMVGR sequence italicized. Polymerase chain reaction (PCR) was performed in a 50 μL reaction volume with 1 μL of DNA template, 1 μL of PFX polymerase (Invitrogen, USA), 5 μL of PFX buffer, 2 μL of dNTP (10 mM), 2 μL of MgSO_4_ and 20 μM of each primer. The PCR reactions were run on a GeneAmp PCR System 9700 (ABI, USA) under the following conditions: 1 cycle of 94°C for 3 min; 30 cycles of 94°C for 30 s, 52°C for 30 s, 72°C for 45 s; 1 cycle of 72°C for 7 min. PCR products were purified using Agencourt AMPure XP kit (Beckman coulter, USA). The DNA quality and concentration of purified PCR products were measured by LabChip GX (Caliper, USA). The qualified samples were then loaded into GS FLX Titanium XLR70 plates (454 Life Sciences/Roche Applied Biosystems, USA) and sequencing was performed by BGI, Shenzhen, China.

### Bioinformatic analysis

The raw data from 454-sequencing were processed using MOTHUR (Version 1.31.2, http://www.mothur.org/). 454-sequencing reads were subjected to subsequent analyses only if they carried the correct forward primer sequences and TCMID sequences, and were ≥200 bp in length. Then the reads were assigned to each sample based on the unique 10-bp TCMID. PyroNoise was used to denoise the sequencing reads, and potential chimeras were identified with UCHIME (Edgar et al., 2011), and removed from subsequent analyses. QIIME (Quantitative Insights Into Microbial Ecology, Version 1.50, http://qiime.sourceforge.net/) was used to assign reads into OTUs (Operational Taxonomic Units) using a 97% identity threshold, and the most abundant sequence from each OTU was selected as the representative sequence for that OTU (Caporaso et al., 2010). Then we used the SILVA database (v108, http://www.arb-silva.de/) and the MAARJAM database (http://maarjam.botany.ut.ee/) to identify representative sequences. SILVA database contains the most abundant whole fungal sequences (Pruesse et al., 2007; Quast et al., 2013), while the MAARJAM database contains all published Glomeromycota SSU rRNA gene sequences (Öpik et al., 2010). The reads were assigned to fungal OTUs using the SILVA database, and those that were identified as Glomeromycota sequences were further assigned to VT (virtual taxa) using the MAARJAM database. We performed a BLAST search to assign sequence reads against the databases with the following criteria: the sequence similarity is ≥97%; the alignment length is no more than 10 bp shorter than the shorter one of the query (sequence reads) and subject (reference database sequence); the BLAST *e*-value is < 1*e*^−10^. For phylogenetic analysis, the representative sequences (the most abundant sequence of one VT) of Glomeromycota VT were aligned using the MAFFT multiple sequence alignment program (version 7, http://mafft.cbrc.jp/alignment/software/, Katoh et al., 2002). A neighbor-joining analysis of all the AMF sequences was performed based on the alignment with TOPALi v2.5 (F84 model with gamma substitution rates and bootstrapping over 100 runs, Milne et al., 2004). To better understand the major AMF species associated with citrus samples, the networks analyses of the AMF species annotated with MAARJAM database among different habitats and scion/rootstock genotypes were created. The networks were processed with the QIIME pipeline and viewed by Cytoscape 3.0 (Cline et al., 2007).

### Statistical analyses

To compare α diversity (within-sample diversity or estimate of species richness) between different samples, Simpson Index, Shannon Index, Observed species (Sobs) and Chao1 Index were used. QIIME was utilized to calculate the AMF diversity (Shannon Index and Simpson Index) and richness (Observed species and Chao1 Index) indices of different samples (Lin et al., 2012). R software (version 2.15.3) was used to plot the rarefaction curve based on the indices of observed species. In order to analyze the influence of different impact factors (such as habitats and host genotypes) on citrus AMF community structure, Principal component analysis (PCA) was performed based on the OTU abundance of each sample (Lin et al., 2012). R software (version 2.15.3) was used to plot the PCA map of all relevant samples or a subgroup of such samples. The Metastats (http://metastats.cbcb.umd.edu/) was used for significance test conversions (White et al., 2009).

## Results

### *Glomus* dominates AMF colonizing citrus root

**Figure 1:**
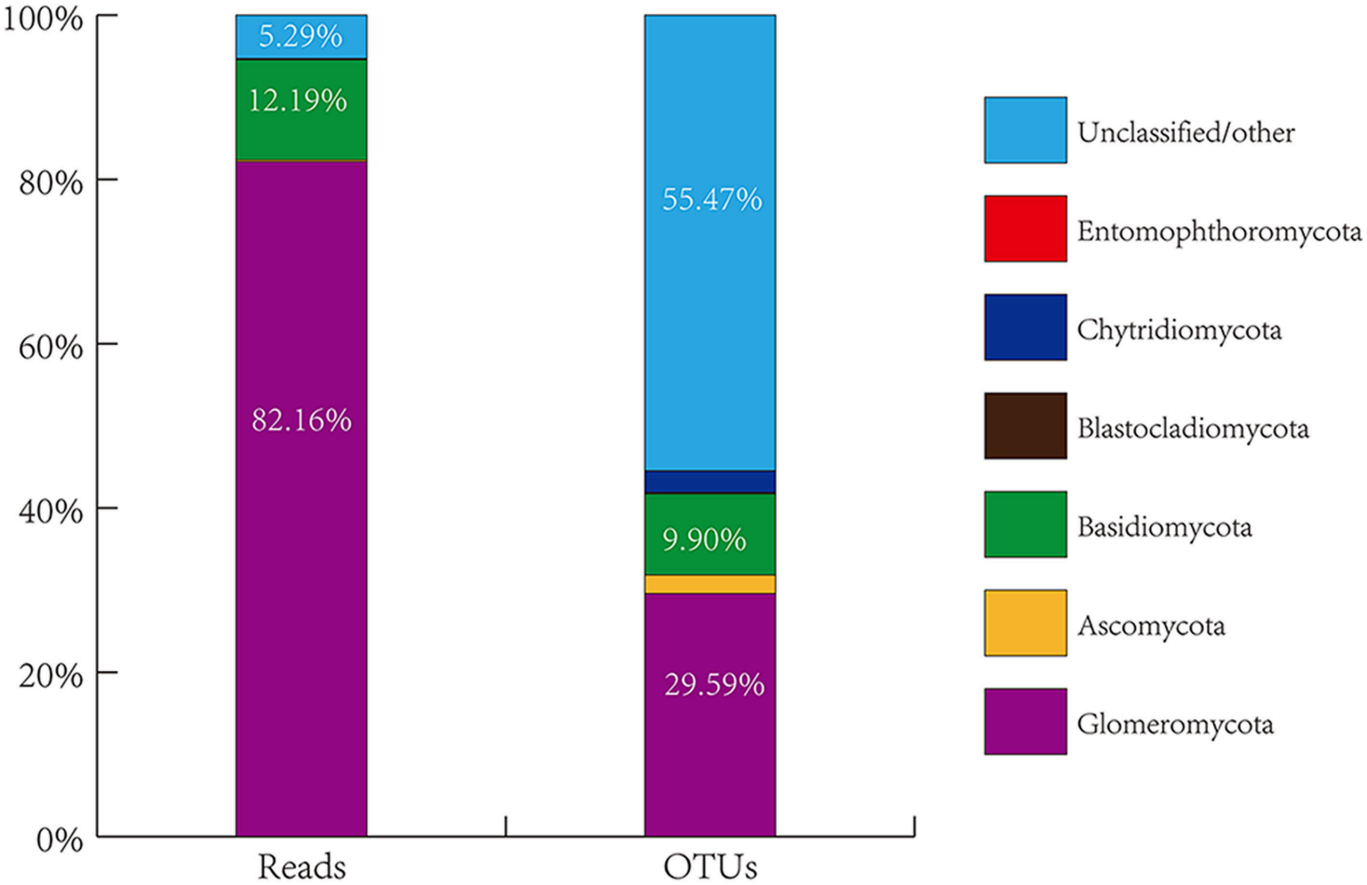
**Proportional distribution of total sequence reads and generated OTUs grouped by phyla of fungi from all citrus root samples through blasting against the SILVA database**.

The overall genetic diversity of AMF colonizing citrus roots is not known, nor is the impact of habitats and host plant genotypes on AMF community structures. To explore these areas, we collected roots of citrus trees grown in eight geographical locations in China. A total of 77 root DNA samples from citrus trees of 23 different genotypes or scion/rootstock combinations (i.e., sample groups) grown under different conditions (For details, see Table S1 and Figure S1, Supporting information) were prepared and used for PCR amplification of the 18S small subunit (SSU) rRNA gene fragment using a pair of largely AMF-specific primers AMV4.5NF/AMDGR. The PCR fragments were purified and subjected to 454-high throughput pyrosequencing. A total of 1,590,218 raw sequence reads were produced. Of which, 7,43,630 were considered to be effective reads (i.e., reads that contain the correct TCMID sequence and the forward primer sequence with a length of ≥200 bp). The effective reads were then assigned to the sequences deposited in SILVA database (v108, http://www.arb-silva.de/), resulting in seven distinct taxonomic groups based on the amplified SSU rRNA gene sequences (Figure 1, Table S2, Supporting information). As expected, the most dominant phylum identified is Glomeromycota to which 6,10,942 effective reads were assigned, accounting for 82.16% of the total effective reads (Table S2). This demonstrates the effectiveness of the AMV4.5NF/AMDGR primer pair in preferential amplification of AMF sequences. Similar to previous studies (Öpik et al., 2009; Lumini et al., 2010; Lin et al., 2012), we also detected some non-AMF sequences that belong to Basidiomycota (90,671 effective reads, accounting for 12.19% of the total effective reads), Ascomycota (1667, 0.22%), Chytridiomycota (953, 0.13%), Blastocladiomycota (66, 0.01%), or Entomophthoromycota (4, nearly 0.00%). A small proportion of amplified sequences (39,327, 5.29%) could not be unambiguously assigned to any of the above phyla (Table S2). To better assess the genetic diversity of the amplified fungal sequences, the 7,43,630 effective reads were clustered into operational taxonomic units (OTU) based on ≥97% sequence similarity, resulting in a total of 3474 OTUs. Among them, 1028 OTUs belong to Glomeromycota (29.59% of the total OTUs), 344 to Basidiomycota (9.90%), 78 to Ascomycota (2.25%), 91 to Chytridiomycota (2.62%), 5 to Blastocladiomycota (0.14%), 1 to Entomophthoromycota (0.03%) and 1927 to the unclassified group (55.47%) (Figure 1, Table S2, Supporting information).

**Figure 2:**
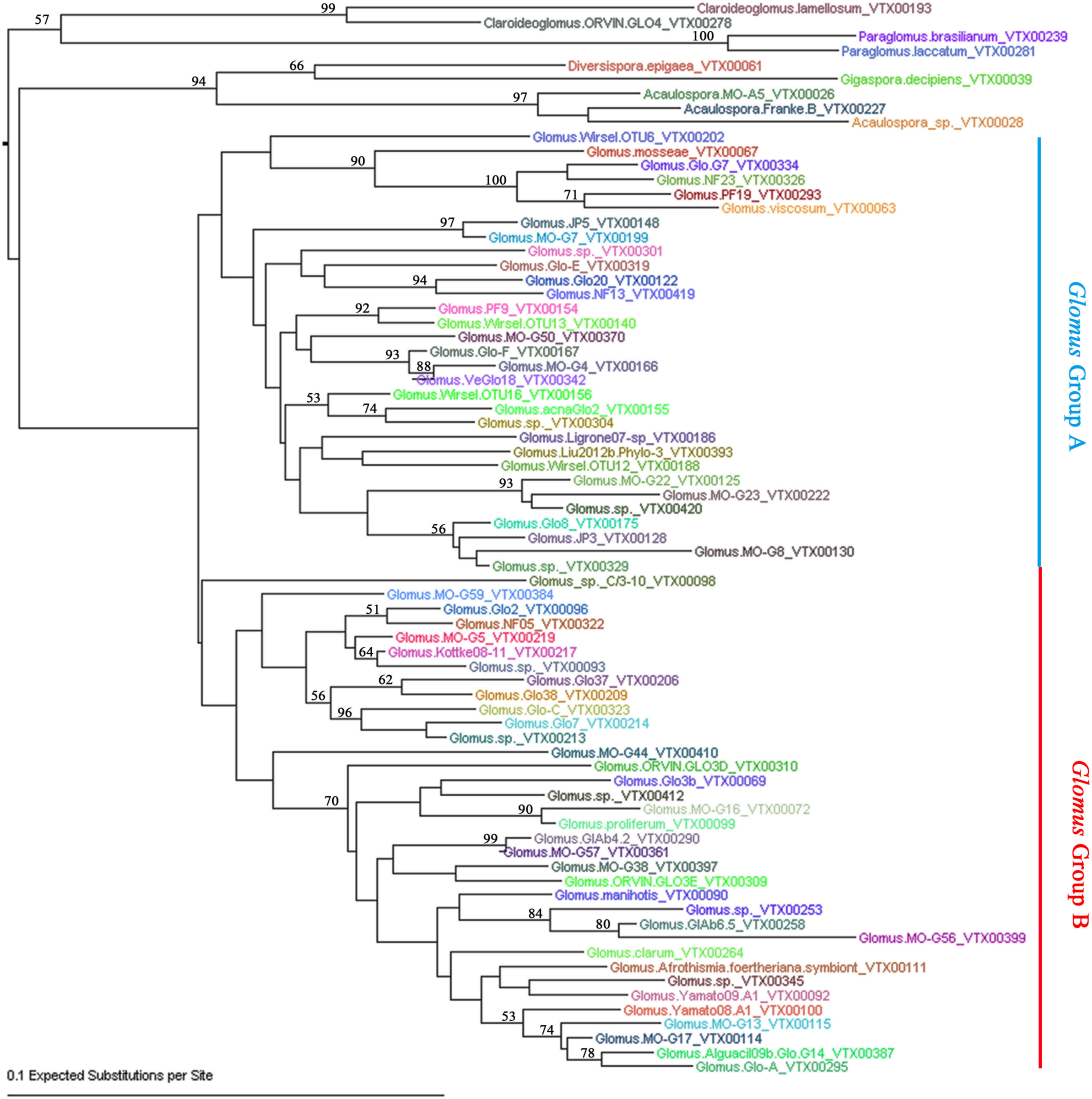
**Neighbor-joining phylogenetic tree of AMF species detected in citrus roots**. The F84+Gamma nucleotide substitution model was used and bootstrap values of >50 are shown.

To further define the AMF sequence diversity, all the effective reads assigned to AMF were aligned against the AMF sequences deposited in MAARJAM database (http://maarjam.botany.ut.ee/), which resulted in 80 AMF virtual taxa (VT). Because five of the 80 VT each has only one effective read, they may be sequence artifacts (Öpik et al., 2009; Hiiesalu et al., 2014) and were excluded from subsequent analyses. Thus, a total of 75 AMF VT (corresponding to 5,27,394 effective reads and 70.92% of total effective reads) were identified from our 77 citrus root samples, indicating a high-level of genetic diversity among the AMF colonizing citrus roots. Based on the neighbor-joining analysis, the 75 VT of Glomeramycota could be assigned to six known AMF families: Glomeraceae (66 VT), Acaulosporaceae (3 VT), Paraglomeraceae (2 VT), Claroideoglomeraceae (2 VT), Paraglomeraceae (1 VT), and Gigasporaceae (1 VT) (Figure 2; Table S3). The 66 Glometaceae VT could be further divided into Glomus group A (31 VT) and Glomus group B (35 VT). Not surprisingly, *Glomus*, the sole genus of the Glomeraceae family, absolutely dominates the AMF in citrus roots, as is the case with other plant species (Lin et al., 2012; Hiiesalu et al., 2014; Xiang et al., 2014). Specifically, the 66 *Glomus* VT account for 99.83% (5,26,478) effective sequence reads with the top 10 *Glomus* VT making up two thirds of the total AMF sequence reads, indicating high heterogeneity in abundance within *Glomus* in citrus roots (Table S3). To better understand the major AMF species associated with citrus samples, we also performed networks analyses for the AMF species among different habitats and scion/rootstock genotypes. Among all the 75 AMF species we detected, only *Glomus*. MO-G117_VTX00114 (also known as *Rhizophagus irregularis*) presented in all of the 23 samples. In addition, the AMF species *Glomus*.sp._VTX00213 and *Glomus*. Glo7_VTX00214 presented in 86% (20/23) of the total samples, *Glomus*.NF13_VTX00419 and *Glomus*.Glo8_VTX00175 presented in 91% (21/23) of the total samples. Results also showed that 10 VT (13.33% of total VT) were shared among 8 habitats, and 3 VT (4%) were shared among 14 host genotypes (*Newhall sweet orange*/*Zhique, Mandarin*/*Red tangerine, Ponkan*/*Citrange, Ponkan*/*Poncirus, Poncirus, Citrange, Limec* × *Orange, Poncirus* × *Tangerine, Orange*/*Poncirus, Mandarin*/*Poncirus, Pummelo*/*Poncirus, Mandarin*/*Yuzu, Newhall sweet orange*/*Red tangerine* and *Newhall sweet orange*/*Poncirus*). Three AMF species *Glomus*.NF13_VTX00419 (2nd abundance of all the VT, 65,008 effective reads, 12.33% of the 5,27,394 effective reads assigned in MAARJAM database), *Glomus*.MO-G17_VTX00114 (3rd, 37,932 effective reads, 7.19%) and *Glomus*.Afrothismia.foertheriana.symbiont_VTX00111 (16th, 9320 effective reads, 1.77%) presented in all the 8 habitats and 14 host genotypes (Figure S3).

### Habitats of citrus trees shape AMF community structures

To assess the influence of a habitat, i.e., the living environment (which includes soil, moisture, temperature, light, and agricultural practices, etc.) where citrus trees are grown, on AMF richness and diversity, we first checked if the sequencing depth of our samples is sufficient to allow a meaningful evaluation. As shown in Figure S4, most rarefaction curves based on observed species (Sobs) from the 77 samples reached a plateau, indicating a sufficient sequencing depth for species richness assessment. Subsequently, we estimated the genetic diversity (α) and species richness for the AMF from the 77 samples collected at eight different geographical locations using four different indices, namely the Simpson and the Shannon Index (for estimation of genetic diversity, which deals with the number of genetic differences contained in a given AMF species), and the Sobs (Observed species) and the Chao1 index (for estimation of species richness, which deals with the number of individual AMF species and their relative abundance in a given habitat) (Lin et al., 2012). As shown in Table 1, there are significant differences (*P* < 0.01) among the samples collected from different locations based on the four indices, which show similar patterns with higher values reflecting higher diversity or richness except for the Simpson index (which is in the reverse order), suggesting that the genetic diversity as well as species richness of the AMF colonizing citrus roots are significantly influenced by habitats. Among the eight locations, Chengdu is ranked the highest both in terms of genetic diversity and species richness for the AMF identified, followed by Hanzhong and Wuhan, whereas Danjiangkou is the lowest (Table 1).

**Table 1:** **The genetic diversity (α) of AMF identified in citrus root samples from eight citrus-producing areas in China**.

**#Alpha**	**Chengdu**	**Danjiangkou**	**Xinfeng**	**Hanzhong**	**Shaoyang**	**Wuhan**	**Xunwu**	**Yiling**
Sobs (^**^)	224.22±9.60	73.33±3.17	81.05±5.81	182.22±6.70	83±13.86	125.10±13.12	83.44±3.72	111.37±11.45
Chao1 (^**^)	274.20±12.32	109.73±14.30	126.92±10.30	221.40±9.41	138.78±30.83	167.47±16.26	115.47±7.32	139.40±11.15
Shannon (^**^)	3.71±0.09	2.39±0.14	2.57±0.16	3.49±0.05	2.68±0.17	2.77±0.16	2.51±0.12	2.57±0.18
Simpson (^**^)	0.057±0.006	0.199±0.048	0.155±0.034	0.057±0.003	0.106±0.015	0.146±0.225	0.163±0.023	0.180±0.032

*The AMF diversity is reflected by Simpson Index, Shannon Index, and AMF richness of Observed species (Sobs), Chao1 Index. Data are means ± SE*.

** *P < 0.01*.

**Figure 3:**
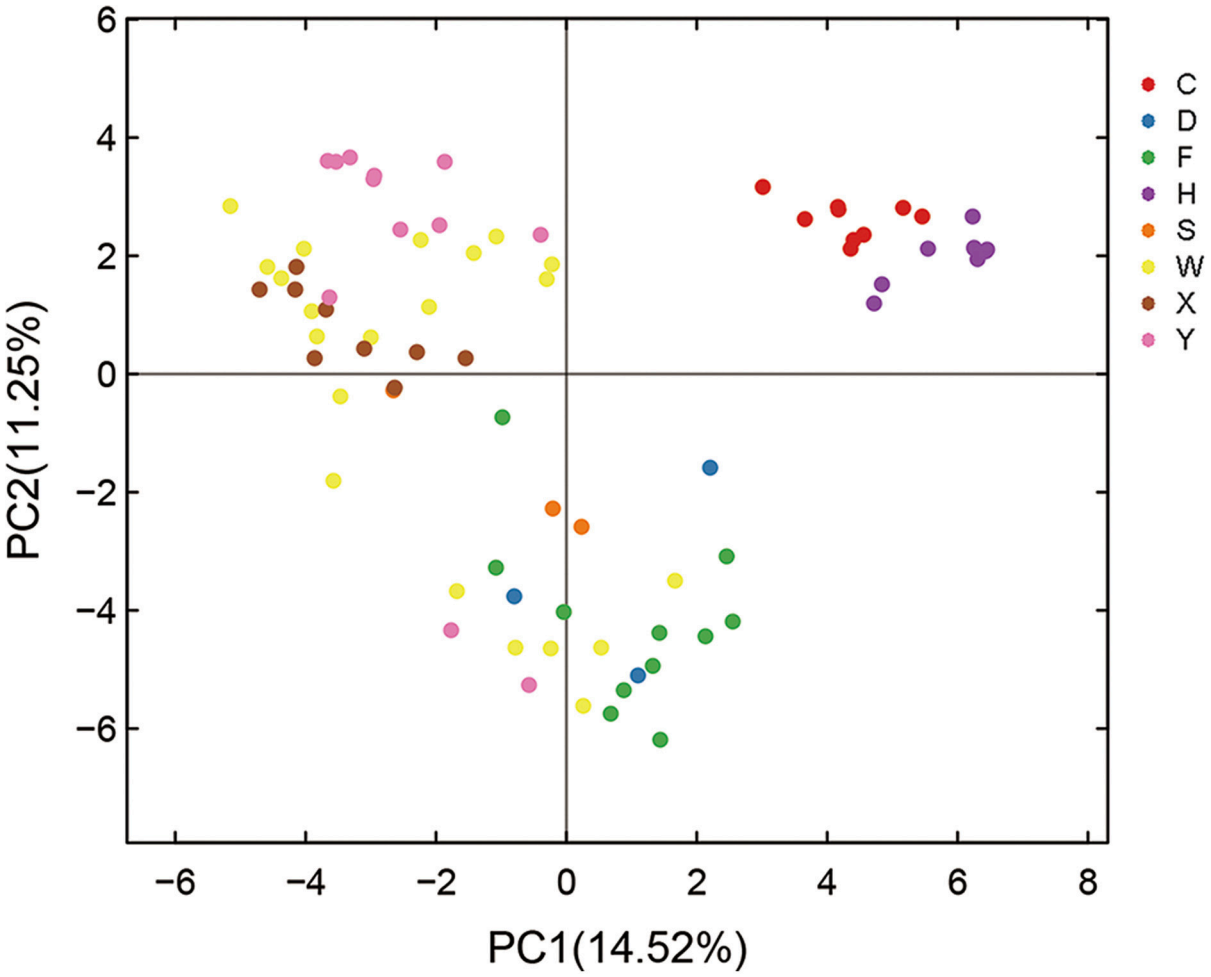
**Principal component analysis (PCA) of variations in citrus AMF community from different habitats**. C means samples collected in Chengdu city; D, Danjiangkou city; F, Xinfeng town; H, Hanzhong city; S, Shaoyang city; W, Wuhan Huazhong Agricultural University; X, Xunwu town; Y, Yiling city.

Principal component analysis (PCA) was performed to further evaluate the effect of habitats on AMF diversity. PCA of the entire set of root samples (i.e., 77 individual samples representing 23 genotypes or scion/rootstock combinations from eight different locations; for details see Table S1 and Figure S1, Supporting information) identified two components that accounted for 25.77% of the total variance, being explained by axis 1 (14.52%) and by axis 2 (11.25%) (Figure 3). Based on this analysis, the AMF community compositions of the 77 root samples can be grouped into three clusters (Figure 3). Cluster I in the upper-right contains all the nine root samples from Hanzhong (violet plots in Figure 3) and nine root samples from Chengdu (red); Cluster II in the upper-left contains ten of the 12 root samples from Yiling (pink), seven of the nine samples from Xunwu (brown) and 13 of the 21 samples from Wuhan (yellow); Cluster III in the bottom contains nine of the 11 samples from Xinfeng (green), two of the three samples from Shaoyang (orange), three samples from Danjiangkou city (blue), and six of the 21 samples from Wuhan (yellow). Thus, based on the PCA analyses, it appears that the AMF community compositions of the root samples collected from Hanzhong, Chengdu, Yiling and Xinfeng were largely distinct from each other and from the rest of the four habitats. Despite that root samples from Xunwu, Shaoyang, Danjiangkou, and Wuhan showed relatively less distinct habitat-specific features in their AMF community compositions, the replicated samples from the same location were still clustered together except for those from Wuhan (which were split into two clusters; for possible reasons, see later section). Because the genotypes of citrus trees sampled vary within or between some habitats (Table S1), these results indicate that habitat has a major impact on citrus AMF community structures, whereas citrus genotypes have relatively less influence on them.

### The scion exerts a greater impact on AMF diversity than the rootstock

**Figure 4:**
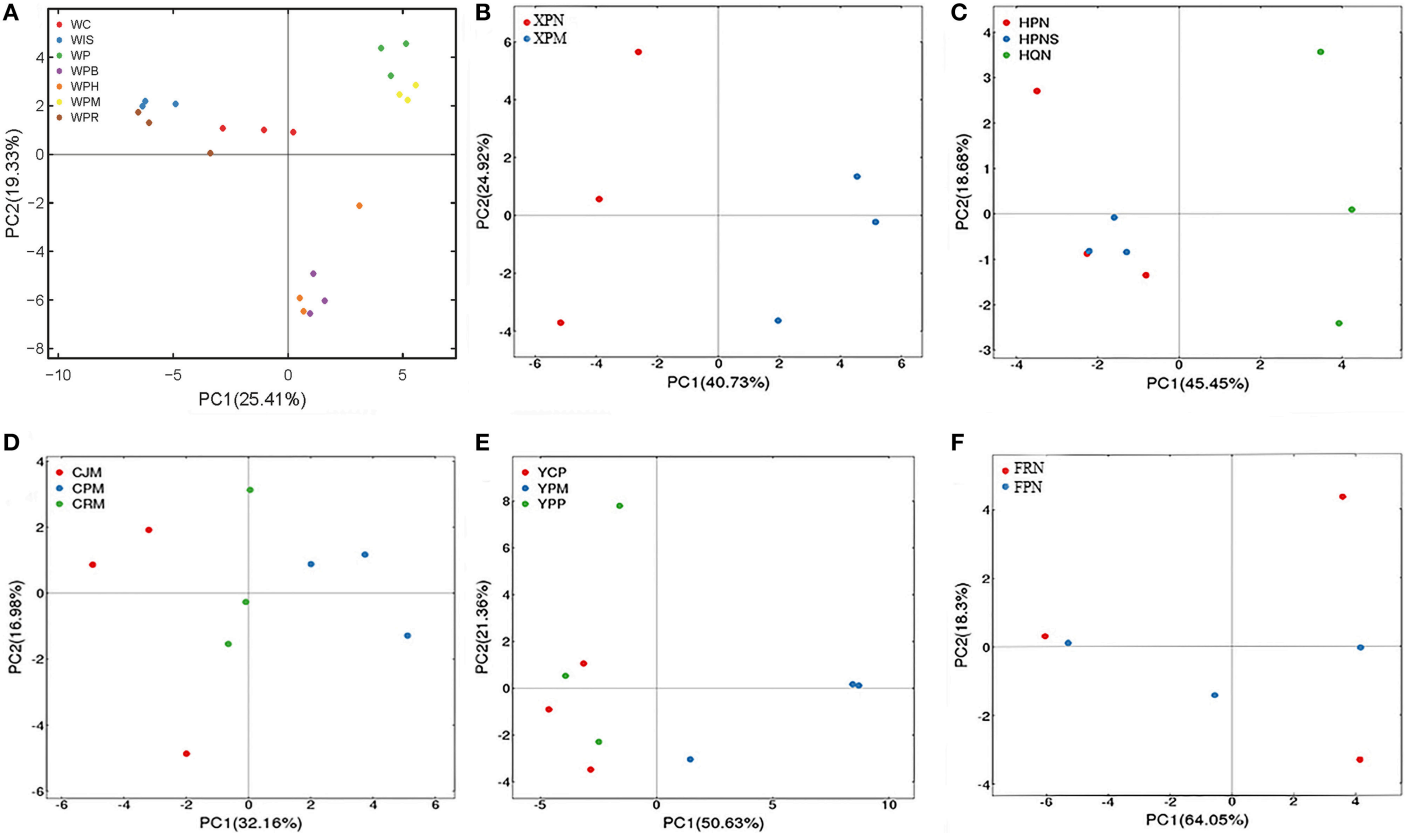
**PCA of variations in citrus AMF community from citrus trees of different genotypes**. **(A)** AMF community of four citrus seedlings, i.e., WC (*Cirange*), WP (*Poncirus*), WIS (*Lime* × *Orange*), and WPR (*Poncirus* × *Red Tangerine*), and three scion/rootstock combinations, i.e., WPH (*Orange*/*Poncirus*), WPM (*Mandarin*/*Poncirus*), WPB (*Pummelo*/*Poncirus*) were separated into three groups based on different scion/rootstock genotypes. **(B)** AMF community of two scion/rootstock combinations (XPN, *Newhall*/*Poncirus*; XPM, *Mandarin*/*Poncirus*) were separated into two groups based on the different scion genotypes. **(C)** AMF community of two scion/rootstock combinations (HPN/HPNS: *Newhall*/*Poncirus*; HQN: *Newhall*/*Zhique*) were separated into two groups based on the rootstock genotypes. **(D)** AMF community of three scion/rootstock combinations (CJM, *Mandarin*/*Yuzu*; CPM, *Mandarin*/*Poncirus*; CRM, *Mandarin*/*Red tangerine*) were separated into three groups based on different rootstock genotypes. **(E)** AMF community of three scion/rootstock combinations (YCP, *Ponkan*/*Cirange*; YPM, *Madarin*/*Poncirus*; YPP, *Ponkan*/*Poncirus*) were separated into two groups based on different scion genotypes. **(F)** AMF communities of the two approach-grafted rootstocks from a single tree (FPN, *Newhall sweet orange*/*Poncirus*; FRN, *Newhall sweet orange*/*Red tangerine*) could not be grouped based on the different rootstock genotypes.

To assess whether genotypes of citrus rootstocks and/or scions influence the AMF community composition, we selected the seven samples (corresponding to 21 replicated plots) from Wuhan for a single PCA analysis based on genotypes of seedlings or scion/rootstock genotype combinations. Specifically, three of these seven samples were collected from citrus trees with distinct scions grafted onto the same type of rootstock, i.e., (i) *Washington navel orange* (*Citrus sinensis*) grafted onto *Poncirus* (*Poncirus trifoliate*) designated *Orange*/*Poncirus*, (ii) *Mandarin* (*Citrus reticulate*) grafted onto *Poncirus*, designated *Mandarin*/*Poncirus*, and (iii) *HB pummelo* (*Citrus grandis*) grafted onto *Poncirus*) designated *Pummelo*/*Poncirus*. The remaining four samples were collected from seed-derived plants (i.e., seedlings) of four different genotypes, i.e., (i) *Poncirus*, (ii) *Citrange* (*Citrus sinensis* × *Poncirus trifoliate*), (iii) an allotetraploid originated from cell fusion of *India lime* and *Sunki orange* designated *Lime* × *Orange*, and (iv) a hybrid originated from hybridization between *Poncirus* and *Red tangerine*, designated *Poncirus* × *Tangerine*. Interestingly, as shown in Figure 4A, these seven samples were clustered into three groups. Group 1 on the upper right side of the PCA plot includes samples from *Poncirus* seedlings (green) or *Mandarin*/*Poncirus* (yellow); Group 2 on the lower right side includes samples from *Pummelo*/*Poncirus* (violet), and *Orange*/*Poncirus* (orange); Group 3 on the upper left side includes samples from seedlings of *Citrange* (red), the *Poncirus* × *Tangerine* hybrid (brown) or the *Lime* × *Orange* allotetraploid (blue), all of which are genetically distinct from *Poncirus*. These results suggest that while the genotypes of the sampled roots clearly influence their AMF community structures, the genotypes of the scions may also impose a significant impact on the AMF community structures of the rootstock as evidenced by distinct clustering between *Mandarin*/*Poncirus* (yellow) and *Pummelo*/*Poncirus* (violet) or *Orange*/*Poncirus* (orange) (Figure 4A). To further evaluate the above inference, we performed PCA analyses for the two samples derived from *Poncirus* rootstocks grafted with two different scions (i.e., *Newhall sweet orange*/*Poncirus* and *Mandarin*/*Poncirus*) in the same orchard at Xunwu (Figure 4B), the three samples collected from Hanzhong [i.e., two from the same *Newhall sweet orange*/*Poncirus* combination in two neighboring orchards, one from *Newhall sweet orange*/*Zhique* (*Citrus ichangensi* × *Poncirus trifoliate*)] (Figure 4C), and the three samples from the same orchard at Chengdu [i.e., *Mandarin*/*Yuzu* (*Citrus junos*)], *Mandarin*/*Poncirus* and *Mandarin*/*Red tangerine* (*Citrus tangerinaHort*) (Figure 4D). The replicate plots of these samples were generally clustered either according to the genotypes of the scions (Figure 4B) or the rootstocks (Figures 4C,D).

To further assess the impact of scion in comparison with rootstock on the genetic diversity of the AMF community, samples from three scion/rootstock combinations, i.e., *Ponkan* (*Citrus reticulata Blanco*)/*Citrange, Ponkan*/*Poncirus*, and *Mandarin*/*Poncirus* collected from the same orchard of Yiling were subjected to PCA analysis. Interestingly, samples from *Ponkan*/*Citrange* and *Ponkan*/*Poncirus* were grouped together, which is distinct from that of *Mandarin*/*Poncirus* (Figure 4E). Given that samples derived from seedlings of *Citrange* or *Poncirus* at Wuhan fell in two distinct groups (Figure 4A), this result suggests that the scion genotype may exert a greater impact on AMF diversity than the rootstock genotype.

To obtain further evidence for this hypothesis, we analyzed the six replicate samples derived from two rootstocks, *Poncirus* and *Red tangerine*, that were approach-grafted to the single scion (*Newhall sweet orange*) (Figure S2, Supporting information) at Xinfeng. As shown in Figure 4F, these samples could not be grouped based on the two rootstock genotypes in a PCA map as it would be predicted if they were seedlings, implying that the scion might have indeed imposed a more significant impact on the AMF community composition in the two rootstocks, thereby resulting in diminishment of the rootstock genotype-exerted impact.

## Discussion

Despite the assumed importance of mycorrhiza in nutrient and water uptake from soil for citrus trees, the overall community structure of AMF colonizing citrus roots in field conditions is largely unknown. In this study, we conducted a comprehensive investigation of the genetic diversity of citrus root-colonizing AMF and found that almost all (99%) AMF identified belong to *Glomus*, highlighting an absolute dominance of this AMF genus in symbiosis with citrus. We further revealed that the citrus AMF community structure is significantly affected by habitats, host genotypes, and tree health status. Most interestingly, our results suggest that the genotype of the scion may exert a greater impact on the AMF community structure than that of the rootstock where the physical root-AMF symbiosis occurs.

The earliest research on citrus AMF diversity was based on spore morphology (Nemec et al., 1981; Vinayak and Bagyaraj, 1990). Characterization of AMF in such studies could only be done at the family level at best (Merryweather and Fitter, 1998). A recent study using DNA-based molecular cloning methods identified AMF of 10 OTUs in citrus roots (Wang and Wang, 2014). In this study, by using the high throughput 454-pyrosequencing technology, we obtained >7,40,000 effective reads falling into 1028 AMF OTUs (SILVA database) or 75 VT (MAARJAM database). We felt confident that this sequence depth and scale should enable identification almost all possible AMF colonizing citrus roots, thus providing a solid basis for subsequent evaluation of potential impact from different factors on citrus AMF community structure. Although similar studies have been reported on AMF recovered from forest (Öpik et al., 2009; Davison et al., 2011; Saks et al., 2014), grassland (Dumbrell et al., 2011; Hiiesalu et al., 2014), farmland (Lin et al., 2012) and farming-pastoral ecotone (Xiang et al., 2014), our work represents the first focused study on AMF colonizing an important perennial woody fruit crop from diverse “habitats”—orchards at eight different geographical locations. The fact that 99.8% of the total effective AMF sequence reads belong to *Glomus* demonstrates the absolute dominance of this AMF genus in symbiosis with citrus. Interestingly, while there appears to be a high intragenic genetic diversity of the *Glomus* AMF (as reflected by the presence of 66 VT under *Glomus*) recovered from citrus roots, there is also apparent enrichment for specific AMF VT (as evidenced by the top 10 VT accounting for two thirds of the total *Glomus* sequence reads). Among the 66 *Glomus* AMF species, networks analyses showed that the most three abundant *Glomus* species presented in most samples (*Glomus*.sp._VTX00213, *Glomus*.MO-G17_VTX00114 and *Glomus*.NF13_VTX00419 presented in 86, 91, and 100% of the total samples, respectively), which could be considered as the dominant AMF species in citrus. Thus, these results depict an overall “landscape” of citrus-AMF association in agricultural settings in major citrus-production areas in China and should serve as a guide for future exploitation of major citrus-adapted AMF VT as potential bio-fertilizer.

It is conceivable that any root microbiota may be shaped not only by host genotypes but also by the habitat environment where the symbiosis is accommodated. Indeed, several studies have shown that AMF community composition could be significantly influenced by soil type, land-use intensity, fertilization scheme, geographic location and vegetation status of the study areas (Landis et al., 2004; Lumini et al., 2010; Davison et al., 2012; Lin et al., 2012; Hiiesalu et al., 2014; Jansa et al., 2014; Xiang et al., 2014). Likewise, genetic diversity of bacterial in *Arabidopsis* is also significantly affected by soil types (Bulgarelli et al., 2012; Lundberg et al., 2012). Thus, it is not surprising that the majority of our root samples were clustered in a location-specific manner despite the genotypic differences in either the rootstocks or the scions involved (Figure 3). This observation suggests that the habitat environment including soil types, climate, orchard management etc. has a dominant impact on the AMF community structure over host genotypes. Since all the eight locations have a similar humid subtropical monsoon climate, it seems that differences in climate could not explain the distinct clustering of AMF from different locations. Instead, soil types or conditions may be a major determinant of the AMF community structures in most if not all of our root samples. We thus collected basic information about soil for the orchards at all the eight graphical locations (Table S4). Interestingly, soil from Chengdu and Hanzhong had the highest pH value (~7.4) and a medium level of organic matter content (~1.6%), whereas soil from Yiling and Xunwu was most acidic (pH 4.8) and had the highest organic matter content (2.5%) (Table S4), coinciding with the clustering of the community structures of AMF sampled from these locations. This suggests that soil pH and/or organic matter content may be major factors associated with a given habitat that influence community structure of AMF colonizing citrus roots.

Citrus, like many other fruit crops, is vegetatively propagated by grafting for commercial production. For example, the vast majority of *Sweet orange* and *Mandarin* cultivars as scion are grafted onto *Poncirus* as rootstock, which is a relative of Citrus known to possess resistance to multiple biotic and abiotic stresses. However, to our knowledge, whether and how much the scion could influence the AMF in the rootstock remains an interesting open question. The deliberate selection of citrus trees with different scion/rootstock combinations in several orchards in this study offered us a unique opportunity to examine the impact of the scion vs. the rootstock on the community structure of the AMF colonizing the rootstock that is genetically different but metabolically integrated with the scion. Given the fact that it is the root that directly interacts with the AMF, we originally thought that the genotype of the scion may have a much weaker, if any at all, impact on the AMF community structure compared with the rootstock. Unexpectedly, our results based on reciprocal scion/rootstock combinations, namely, different scion genotypes on the same rootstock genotype and the same scion genotype on different rootstock genotypes, generally support an inference that the scion genotype may exert even a greater impact than the rootstock genotype on the AMF community structure (Figure 4). For example, *Mandarin* and *sweet orange* as scion resulted in distinguishable AMF community structures in their *Poncirus* rootstocks in the same orchard at Wuhan (Figure 4A) and also at Xunwu (Figure 4B), whereas *sweet orange* as scion grafted to *Poncirus, red tangerine*, or *Yuzu* as rootstock produced AMF community structures less distinguishable (Figure 4D). More convincing evidence came from (i) the grouping of *Ponkan*/*Citrange* and *Ponkan*/*Poncirus* samples together but away from those of *Mandarin*/*Poncirus* (Figure 4E) in the same orchard at Yiling and (ii) the diminishment of differences in the AMF community composition between *Poncirus* and *Red tangerine* as two separate rootstocks of the same scion (sweet orange) (Figure 4F).

It is well-known that rootstocks could influence many agronomic traits of their scions including stress tolerance and fruit quality (Haroldsen et al., 2012; Marguerit et al., 2012; Benjamin et al., 2013). However, it is poorly understood how scions might impact the physiology of their rootstocks. Our observation with regard to the impact of the scion genotype on the community structure of the AMF in the rootstock strongly suggests that the scion, or the aboveground of any plant by extrapolation, could impose significant influence on the biological activity and habitat-adaptability of the root (stock). But how could the scion of a grafted tree possibly exerts an even greater impact than the rootstock on the AMF community in the rootstock? One likely explanation is that primary and secondary metabolites that are synthesized by the aboveground shoots and leaves, and transported to the roots, could function as important chemical cues for AMF to engage selective symbiosis with the host plants (Broeckling et al., 2008; Micallef et al., 2009; Kiers et al., 2011). Another possibility is that a regulatory mechanism similar to shoot-controlled root nodulation and symbiosis with rhizobia (Krusell et al., 2002; Nishimura et al., 2002; Bisseling and Scheres, 2014), might also operate in plants for modulating mycorrhizal symbiosis, rendering an explanation for a greater impact of the scion (shoots) on the community structure of the AMF than the rootstock in some cases.

Taken together, this study represents a comprehensive analysis of the community composition and structure of AMF in symbiosis with citrus roots. Not only have we identified all the major AMF species that form symbiosis with citrus but we also revealed the impact of habitats and host (particularly the scion) genotypes on the AMF community composition and structure. Therefore, this study should lay a solid foundation for future research on and exploitation of AMF for enhancing citrus production.

## Author contributions

Zhiyong Pan and Shunyuan Xiao designed the research; Fang Song performed the experiment and data analysis; Fang Song and Zhiyong Pan wrote the manuscript; Fang Song, Fuxi Bai, and Jianyong An collected the samples; Zhiyong Pan, Shunyuan Xiao, Jihong Liu, Ton Bessling, and Xiuxin Deng guided the research. All authors had read, edited and approved the final manuscript.

### Conflict of interest statement

The authors declare that the research was conducted in the absence of any commercial or financial relationships that could be construed as a potential conflict of interest. The handling editor declared a shared affiliation, though no other collaboration, with one of the authors and states that the process nevertheless met the standards of a fair and objective review.

## Supplementary material

### Accessibility

The raw pyrosequencing data are accessioned at the NCBI short read archive (Accession no.SRP060279) The final taxon table with the read count data was shown in Table S5 (Supporting information).

## Abbreviations

AMF: Arbuscular mycorrhizal fungi
VT: virtual taxa
PCA: Principal component analysis
SSU: small subunit
SE: standard error.
