# Supplementary material for: The Scion/Rootstock Genotypes and Habitats Affect Arbuscular Mycorrhizal Fungal Community in Citrus
Source: Front Microbiol. 2015 Dec 1;6:1372. doi: 10.3389/fmicb.2015.01372 (PMC4664953; doi:10.3389/fmicb.2015.01372)
Supplement: Supplementary file 6 [file Table6.DOCX]

**Supplementary Material**

**Defining the core arbuscular mycorrhizal fungal community structure and its variation under different habitats, host plant genotypes, and Huanglongbing infection in citrus**

Fang Song^1^, Zhiyong Pan^1^*, Fuxi Bai^1^, Jianyong An^1^, Jihong Liu^1^, Wenwu Guo^1^, Ton Bisseling^2^, Shunyuan Xiao^1,3^, Xiuxin Deng^1^

*Correspondence: Dr. Zhiyong Pan, Huazhong Agricultural University, College of Horticulture and Foretry Sciences, Key Laboratory of Horticultural Plant Biology (Ministry of Education). Shizishan Street, NO.1, Wuhan, 430070, China. [zypan@mail.hzau.edu.cn](mailto:zypan@mail.hzau.edu.cn)

**Supplementary Material Legends**

**Table S1 The distribution, replicated plots numbers and description of the root samples used in this study.**

**Table S2 The total reads and generated OTUs of all the samples grouped by phyla of fungi through blasting against the SILVA database.**

**Table S3 The genetic diversity (α) of AMF identified in samples from the healthy (XPM) and HLB-infected (XPMH) citrus roots samples with the same genotypes of Mandarin/Poncirus in Xunwu.** The AMF diversity is reflected by Simpson Index, Shannon Index, and AMF richness of Observed species (Sobs), Chao1 Index. Data are means ± SE.

**Table S4 Basic information about the eight habitats from which citrus root samples were collected.**

**Table S5 AMF community matrix at species level**

**Fig. S1 The geographic locations of 8 sampling sites (citrus producing areas) in China.**

**Fig. S2 A cartoon illustrating a citrus tree consisting of a single scion (Newhall sweet orange) and two rootstocks (Poncirus and Red tangerine) cultivated via approach-graftinggrafted to the single scion.**
