# Supplementary material for: The Scion/Rootstock Genotypes and Habitats Affect Arbuscular Mycorrhizal Fungal Community in Citrus
Source: Front Microbiol. 2015 Dec 1;6:1372. doi: 10.3389/fmicb.2015.01372 (PMC4664953; doi:10.3389/fmicb.2015.01372)
Supplement: Supplementary file 7 [file DataSheet1.PDF]

Supplementary Material

Defining the core arbuscular mycorrhizal fungal community structure and its variation under different habitats, host plant genotypes, and Huanglongbing infection in citrus

Fang Song[1], Zhiyong Pan[1]*, Fuxi Bai[1], Jianyong An[1], Jihong Liu[1], Wenwu Guo[1], Ton Bisseling[2], Shunyuan Xiao[1,3], Xiuxin Deng[1]

*Correspondence: Dr. Zhiyong Pan, Huazhong Agricultural University, College of Horticulture and Foretry Sciences, Key Laboratory of Horticultural Plant Biology (Ministry of Education). Shizishan Street, NO.1, Wuhan, 430070, China. zypan@mail.hzau.edu.cn

Supplementary Material Legends

Table S1 The distribution, replicated plots numbers and description of the root samples used in this study.

Table S2 The total reads and generated OTUs of all the samples grouped by phyla of fungi through blasting against the SILVA database.

Table S3 The genetic diversity (α) of AMF identified in samples from the healthy (XPM) and HLB-infected (XPMH) citrus roots samples with the same genotypes of Mandarin/Poncirus in Xunwu. The AMF diversity is reflected by Simpson Index, Shannon Index, and AMF richness of Observed species (Sobs), Chao1 Index. Data are means ± SE.

Table S4 Basic information about the eight habitats from which citrus root samples were collected.

Table S5 AMF community matrix at species level

Fig. S1 The geographic locations of 8 sampling sites (citrus producing areas) in China.

Fig. S2 A cartoon illustrating a citrus tree consisting of a single scion (Newhall sweet orange) and two rootstocks (Poncirus and Red tangerine) cultivated via approach-graftinggrafted to the single scion.

Fig. S3 Networks analyses of all the AMF species under different habitats (A) and scion/rootstock genotypes (B). The numbers in the squares indicated the 75 AMF species as shown in Table S3. AMF species highlighted in green indicated that this species was shared by all the variables (habitats, scion/rootstock genotypes). C means samples collected in Chengdu city; D, Danjiangkou city; F, Xinfeng town; H, Hanzhong city; S, Shaoyang city; W, Wuhan Huazhong Agricultural University; X, Xunwu town; Y, Yiling city.

Fig. S4 Rarefaction curves of all the samples measured by observed species.
