# Supplementary figures and images for: The Scion/Rootstock Genotypes and Habitats Affect Arbuscular Mycorrhizal Fungal Community in Citrus
Source: Front Microbiol. 2015 Dec 1;6:1372. doi: 10.3389/fmicb.2015.01372 (PMC4664953; doi:10.3389/fmicb.2015.01372)

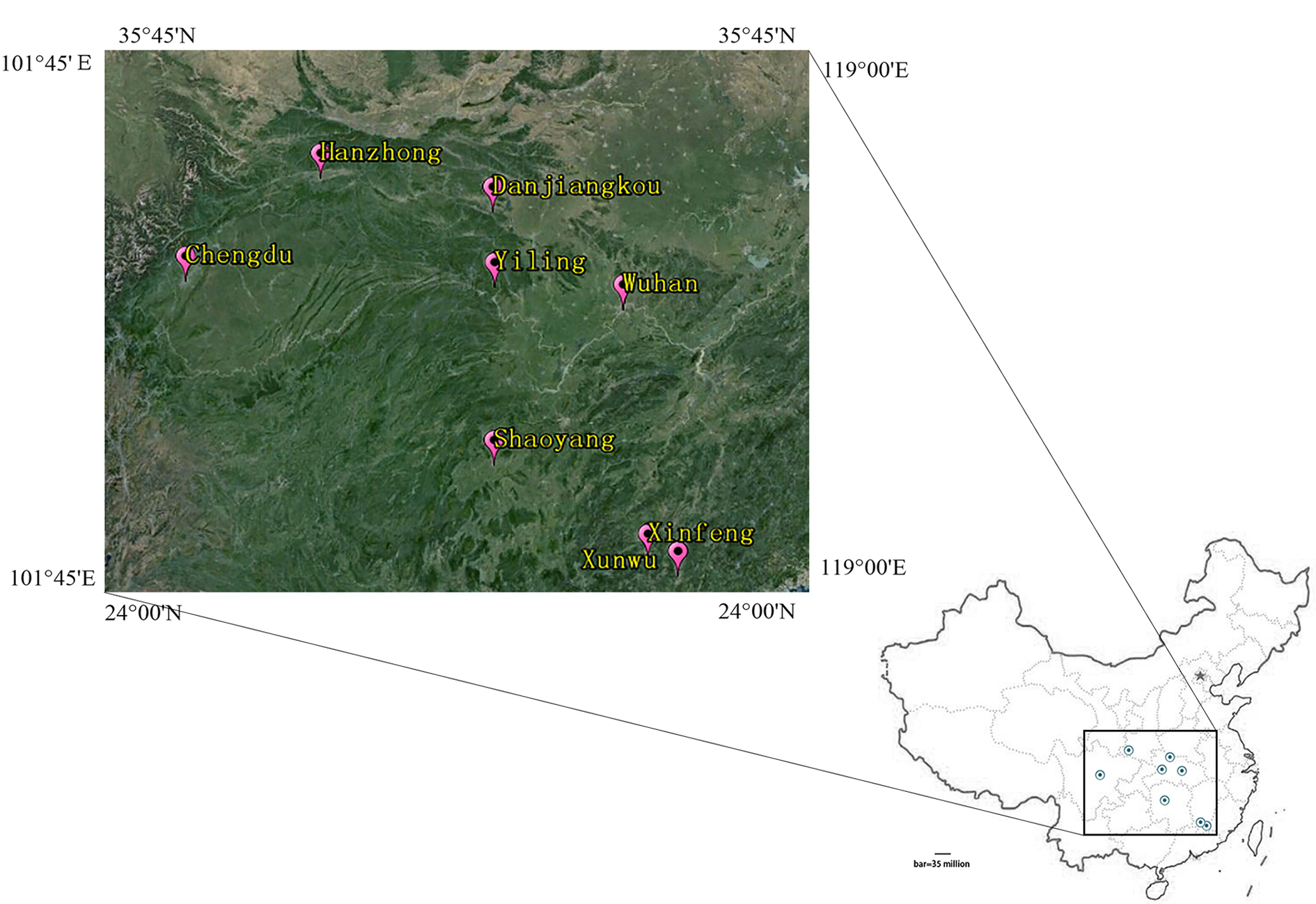

Supplement: Supplementary file 8 [file Image1.TIF]

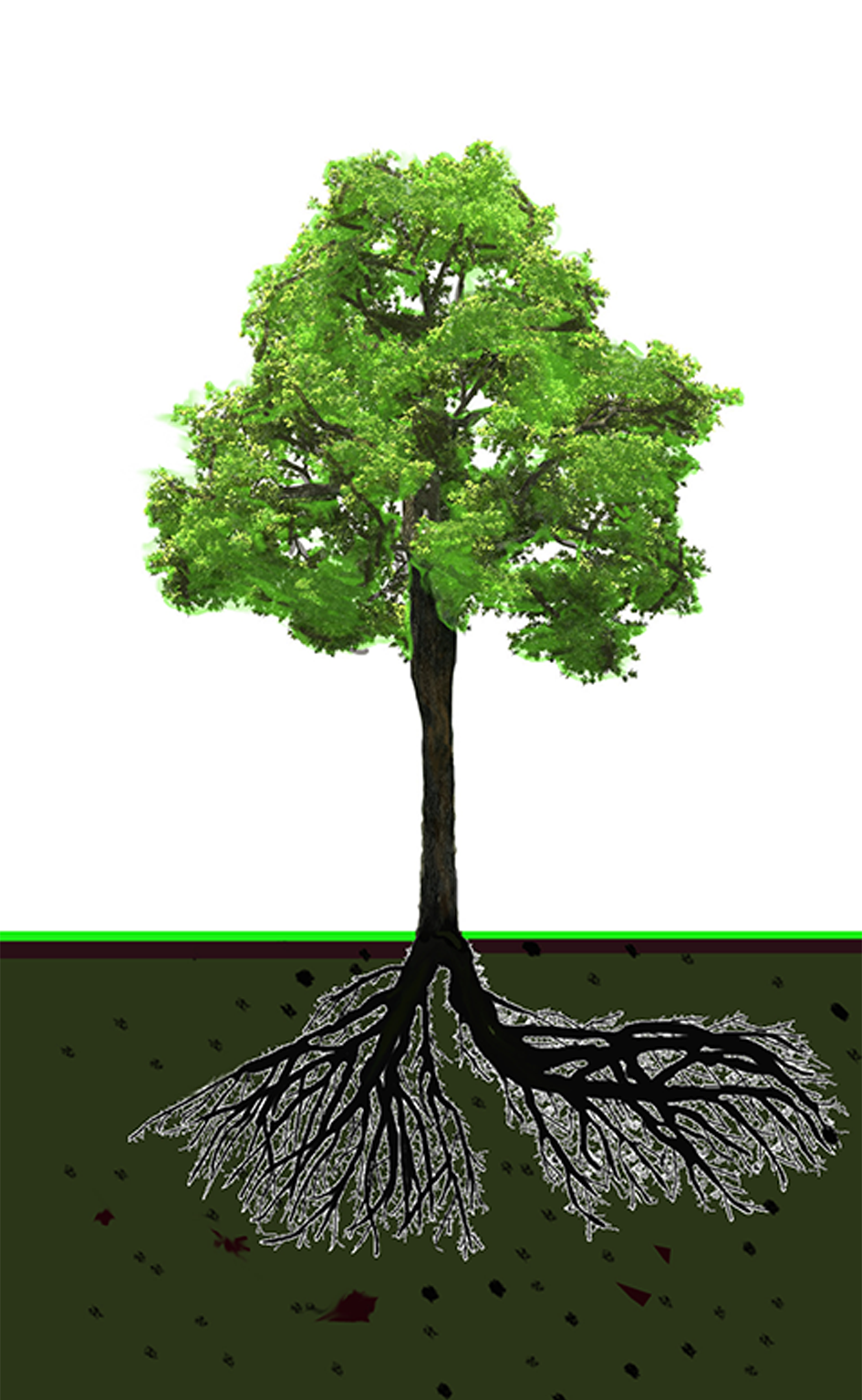

Supplement: Supplementary file 9 [file Image2.TIF]

B

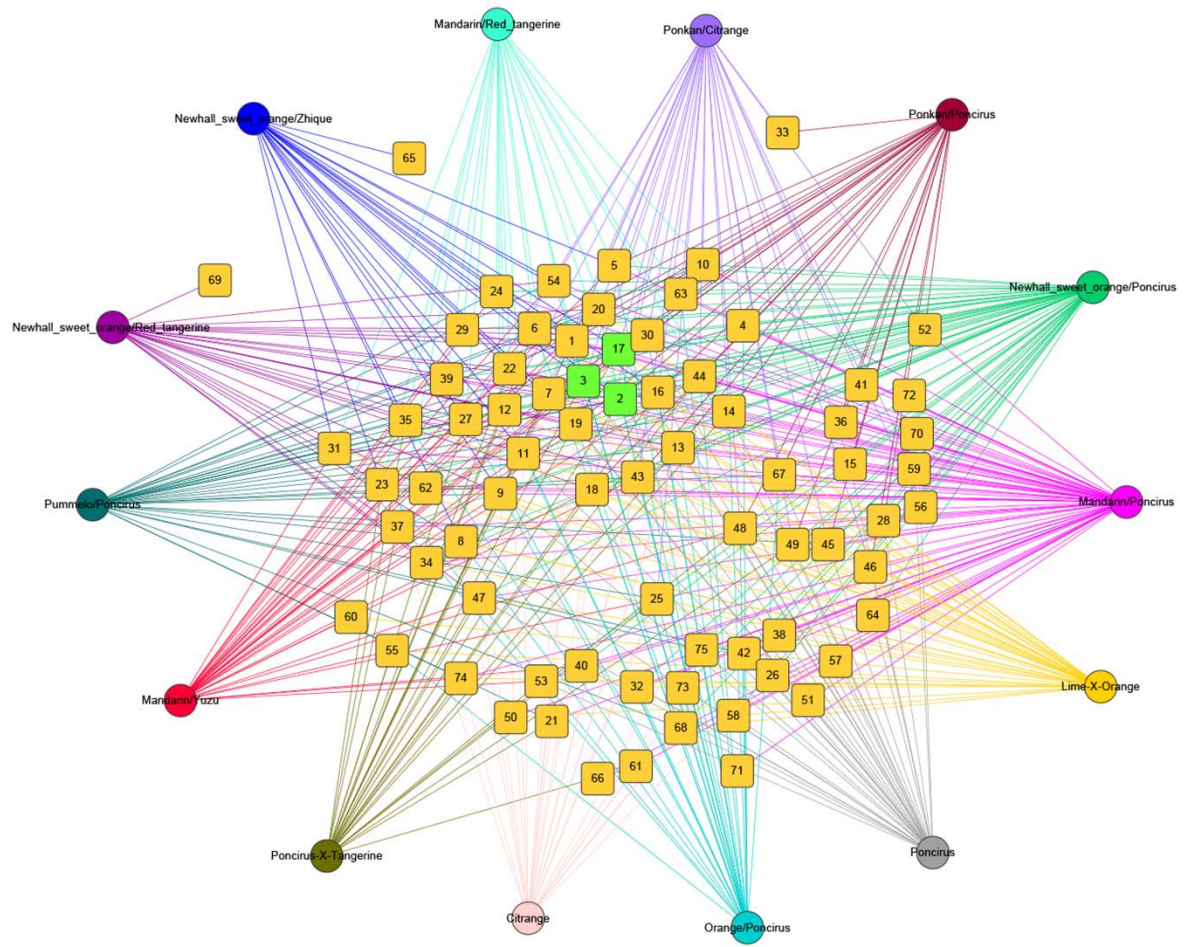

Supplement: Supplementary file 10 [file Image3.PDF]

**Fig. S4 Rarefaction curves of all the samples measured by observed species.**

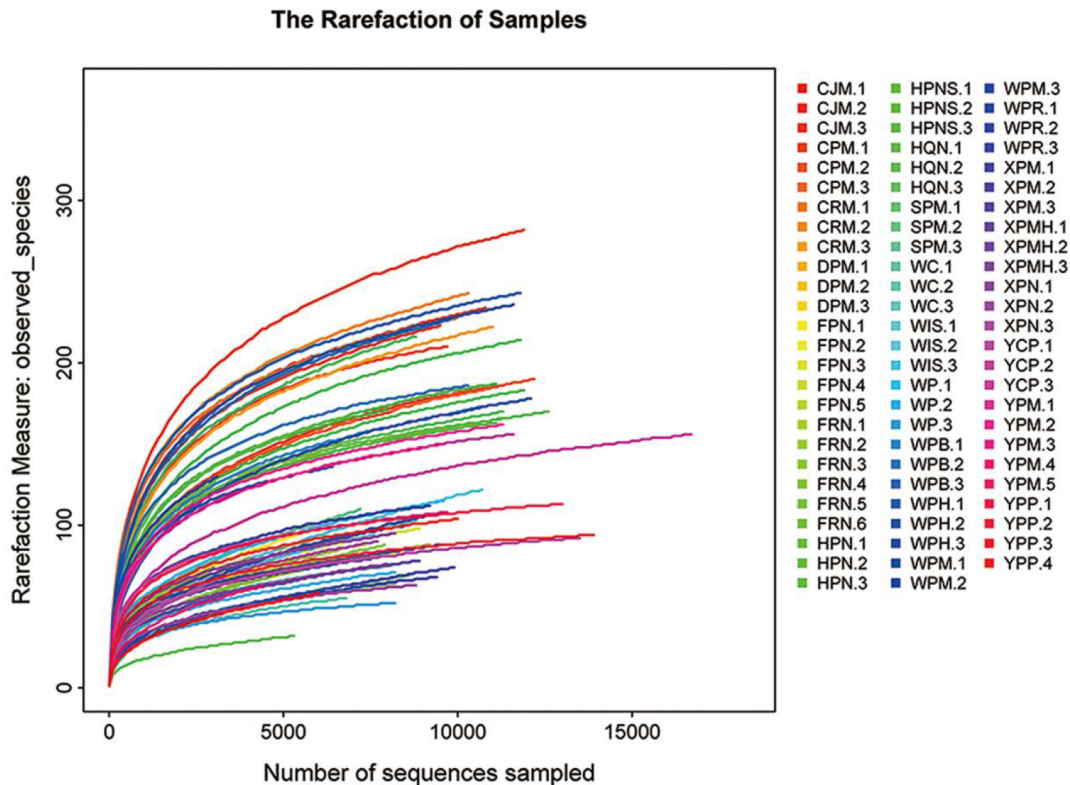

Supplement: Supplementary file 11 [file Image4.PDF]
